# Optimal folic acid dosage in lowering homocysteine: Precision Folic Acid Trial to lower homocysteine (PFAT-Hcy)

**DOI:** 10.1007/s00394-024-03344-8

**Published:** 2024-03-13

**Authors:** Xiao Huang, Huihui Bao, Congcong Ding, Junpei Li, Tianyu Cao, Lishun Liu, Yaping Wei, Ziyi Zhou, Nan Zhang, Yun Song, Ping Chen, Chongfei Jiang, Liling Xie, Xianhui Qin, Yan Zhang, Jianping Li, Ningling Sun, Genfu Tang, Xiaobin Wang, Hong Wang, Yong Huo, Xiaoshu Cheng, for the Precision Folic Acid Trial to lower homocysteine (PFAT-Hcy) Trial Investigators

**Affiliations:** 1 Department of Cardiology, The Second Affiliated Hospital, Jiangxi medical College, Nanchang University, Nanchang, China; 2 Center for Prevention and Treatment of Cardiovascular Diseases, The Second Affiliated Hospital of Nanchang University, Nanchang, China; 3 Biological Anthropology, University of California Santa Barbara, Santa Barbara, CA USA; 4 Institute of Biomedicine, Anhui Medical University, Hefei, China; 5 Graduate School at Shenzhen, Tsinghua University, Shenzhen, China; 6 Beijing Advanced Innovation Center for Food Nutrition and Human Health, College of Food Science and Nutritional Engineering, China Agricultural University, Beijing, China; 7 Department of Cardiology, Peking University First Hospital, Beijing, China; 8 College of Pharmacy, Jinan University, Guangzhou, China; 9 The Department of Nephrology, The University of Hongkong-Shenzhen Hospital, Shenzhen, China; 10 National Clinical Research Study Center for Kidney Disease, The State Key Laboratory for Organ Failure Research, Renal Division, Nanfang Hospital, Southern Medical University, Guangzhou, China; 11 Department of Hypertension, Heart Center, Peking University People’s Hospital, Beijing, China; 12 School of Health Administration, Anhui Medical University, Hefei, China; 13 Department of Population, Family and Reproductive Health, Johns Hopkins University Bloomberg School of Public Health, Baltimore, USA; 14 Centers for Metabolic Disease Research, Cardiovascular Research and Thrombosis Research, Temple University, Lewis Katz School of Medicine, Philadelphia, PA USA

**Keywords:** Folic acid, Homocysteine, MTHFR *C677T* genotypes, Optimal dosage

## Abstract

**Background:**

While folic acid (FA) is widely used to treat elevated total homocysteine (tHcy), promoting vascular health by reducing vascular oxidative stress and modulating endothelial nitric oxide synthase, the optimal daily dose and individual variation by MTHFR *C677T* genotypes have not been well studied. Therefore, this study aimed to explore the efficacy of eight different FA dosages on tHcy lowering in the overall sample and by *MTHFR C677T* genotypes.

**Methods:**

This multicentered, randomized, double-blind, controlled clinical trial included 2697 eligible hypertensive adults with elevated tHcy (≥ 10 mmol/L) and without history of stroke and cardiovascular disease. Participants were randomized into eight dose groups of FA combined with 10 mg enalapril maleate, taken daily for 8 weeks of treatment.

**Results:**

The intent to treat analysis included 2163 participants. In the overall sample, increasing FA dosage led to steady tHcy reduction within the FA dosing range of 0–1.2 mg. However, a plateau in tHcy lowering was observed in FA dose range of 1.2–1.6 mg, indicating a ceiling effect. In contrast, FA doses were positively and linearly associated with serum folate levels without signs of plateau. Among MTHFR genotype subgroups, participants with the TT genotype showed greater efficacy of FA in tHcy lowering.

**Conclusions:**

This randomized trial lent further support to the efficacy of FA in lowering tHcy; more importantly, it provided critically needed evidence to inform optimal FA dosage. We found that the efficacy of FA in lowering tHcy reaches a plateau if the daily dosage exceeds 1.2 mg, and only has a small gain by increasing the dosage from 0.8 to 1.2 mg.

**ClinicalTrials.gov Identifier:**

NCT03472508 (Registration Date: March 21, 2018).

**Supplementary Information:**

The online version contains supplementary material available at 10.1007/s00394-024-03344-8.

## Introduction

Folate, a water-soluble vitamin, includes endogenous food folate and its synthetic form, folic acid (FA) [1]. Previous studies have demonstrated the effectiveness of FA supplementation in preventing neural-tube defects (NTD) in newborns. Since 1996, nations such as the USA and Canada have introduced mandatory FA fortification of white flour [2]. Such regulations are currently widely in place in 53 countries worldwide [3]. However, despite the knowledge that FA supplementation reduces the risk of NTD such as spina bifida during the periconceptional period [4, 5], its efficacy on cardiovascular and cerebrovascular disease prevention remains controversial. A meta-analysis we conducted earlier using randomized trial data showed that in countries without FA fortification, FA supplementation considerably lowers the risk of stroke, whereas it presents limited benefit in fortified countries [6]. Furthermore, folic acid therapy in low doses, alike daily intake, and dietary fortification, can enhance vascular function by reducing vascular oxidative stress and modulating nitric oxide synthase [7]. Baseline folate differs by various factors such as fortification regulations and is particularly associated with homocysteine level [8].

Recent studies have determined elevated plasma total homocysteine (tHcy) level to be well-established modifiable risk factors for cerebral–cardiovascular disease [9–11]. Elevation of circulating tHcy concentrations is also closely related with numerous nutritional, hormonal, and genetic factors, and thus is associated with particular pathological conditions. Given that the majority of the body’s tHcy participates in the re-methylation process through one-carbon metabolism, which requires a folate-derived methyl donor, folic acid therapy has been proposed as a key strategy for lowering tHcy levels.

The primary carbon donor in the re-methylation of tHcy to methionine is 5-methyltetrahydrofolate, which is synthesized by methylenetetrahydrofolate reductase (MTHFR). However, a prevalent MTHFR mutation that replaces alanine with valine can render the enzyme thermolabile and results in increased plasma levels of tHcy. Those with thermolabile MTHFR may require a greater amount of folate to regulate plasma tHcy concentrations [12, 13]. The most compelling evidence supporting the effectiveness of reducing tHcy levels in preventing risk is observed in the China Stroke Primary Prevention Trial (CSPPT), which indicates that a 20% reduction in tHcy led to 7% decrease in the risk of incident stroke and composite cardiovascular disease [14]. Additionally, a subset of the CSPPT showed that the *MTHFR C677T* genotype modified the FA therapeutic effect [15]. With aligned long-term 0.8 mg/day FA therapy, about 30% of individuals with the TT genotype did not reach the folate threshold of ≥ 15 ng/mL, and only about 20% from the TT group achieved a tHcy level below10 μmol/L at the exit visit.

The Chinese population without mandatory FA fortification has a high proportion of insufficient folate intake (> 50%), hyperhomocysteinemia, and the *MTHFR* gene polymorphism [16, 17]. In particular, Chinese patients with hypertension accompanied by elevated tHcy (≥ 10 μmol/L) have a synergistically increased risk of stroke [18, 19]. Thus, stroke prevention strategies that target the lowering of tHcy levels in hypertensive patients are essentially needed. In addition, a post-mortem analysis by the CSPPT showed that in people with low levels of folic acid, a daily intake of 0.8 mg of folic acid was associated with a lower risk of malignant tumors associated with stress [20]. However, to date, there is a general lack of consensus on the optimal folic acid supplementation dosage, and the recommended supplementation strategies vary among different countries [21].

An urgent need for further investigation on folic acid therapy strategies, as well as the optimal FA dose required in tHcy lowering among the various *MTHFR* genotypes. Thus, the following folic acid dose titration study, the Precision Folic Acid Trial to lower homocysteine (PFAT-Hcy, ClinicalTrials.gov Identifier: NCT03472508), was designed. This is by far the first and largest folic acid intervention trial in Chinese adults with hypertension and elevated tHcy, a population exposed to higher stroke and cardiovascular disease risks. This paper lays down the essential background and conclusions for future research, which will provide high-quality evidence to inform clinical and public health guidelines on the optimal dose of folic acid for tHcy lowering, while considering individual *MTHFR* genotype.

## Materials and methods

### Study oversight

PFAT-Hcy is a multi-centered, randomized, double-blind, controlled clinical trial, conducted at Wuyuan, Anqing, and Lianyungang, China. The details of the protocol have been published elsewhere [22]. Briefly, inclusion criteria included men and women aged 45–75 years with hypertension, defined as resting seated systolic blood pressure ≥ 140 mmHg and/or diastolic blood pressure ≥ 90 mmHg and/or taking antihypertensive medication, and elevated tHcy (≥ 10 μmol/L) during both the screening and recruitment visits conducted at least 1 day apart. Persons with a history of cardiovascular diseases were excluded, as were persons with any chronic disease that might interfere with folate or homocysteine metabolism (e.g., renal disease, thyroid disease, liver disease). Anyone taking dietary supplements containing B vitamins or compounds within 3 months of study initiation were excluded. Eligible patients were randomly assigned to one of eight doses of FA daily treatment group from 0 to 2.4 mg (0, 0.4, 0.6, 0.8, 1.2, 1.6, 2.0, 2.4 mg). The trial was conducted between August 2017 and October 2018. The primary end point was tHcy lowering. Follow-up visits were completed at the end of the 2nd, 4th, 6th, and 8th week of treatment.

### Ethics approval

This study was approved by the Medical Ethics Committee of the Second Affiliated Hospital of Nanchang University’s Local Ethical Review process (2018–02–2). Written informed consents were provided by all study participants.

### Physical examination, demographic characteristics, and laboratory assays

During the first screening visit, which priors the treatment visit, physical examination was conducted to assess patients’ clinical diagnosis and eligibility for inclusion in the study. Other demographic information and information related to this study’s inclusion/exclusion criteria were also obtained.

Fasting (overnight) venous blood samples (8 mL each) and spot urine samples (10 mL) were collected at baseline and at the end of the 4th and 8th week after the double-blind treatment began. The TaqMan assay was used to detect the MTHFR C677T (rs1801133) polymorphisms using the ABI Prism 7900HT sequence detection system (Life Technologies). Serum folate, vitamin B12, and B6 were measured using a chemiluminescent immunoassay (New Industrial) at a commercial laboratory at both the baseline and exit visit during the run-in period and the double-blind treatment period. Serum fasting lipids and glucose, creatinine, and tHcy were measured using automatic clinical analyzers (Beckman Coulter) at the central laboratory of the Shenzhen Tailored Medical Laboratory at both the baseline and exit visit [23, 24]. While they were not analyzed in this study, these laboratory results will be used in future statistical analyses and in the final report. In addition, serum 5-methyltetrahydrofolate (5-MeTHF), unmetabolized folic acid, S-adenosinemethionine (SAM), and S-adenosinehomocysteine (SAH) were measured for future further analysis.

### Efficacy indicator

The primary efficacy indicator was the percentage decrease in blood tHcy levels by the end of the 8th week from baseline [tHcy percent decrease = (baseline tHcy-end tHcy)/baseline tHcy*100%]. Secondary efficacy indicators include the magnitude of decrease in tHcy by the end of the 8th week from baseline [absolute tHcy reduction (mmol/L) = baseline tHcy-end tHcy] and percentage and magnitude of increase in blood folate levels by the end of the 8th week from baseline.

### Statistical analysis

Based on a previous study, tHcy lowering rate for enalapril folic acid tablet 10/0.8 mg was anticipated to be 11–12%, whereas the control group (enalapril) almost stayed the same [15]. To achieve 80% power while maintaining a type I error rate of 0.00625 for pairwise comparisons among eight groups with Bonferroni correction (0.05/8 = 0.00625), a minimum of 165 participants per group would be required. Further considering potential cases of withdrawal, shedding, and low compliance (10–20%), the total sample size for this study was 1600 cases with 200 participants per group. Based on all dose groups, 0.8 mg is the intermediate dose group, and the calculated sample size can meet the research hypothesis. This trial, with a sample size of 2163 eligible participants in the final analysis, was adequately powered to address the primary study hypothesis.

Means (SD) and proportions were calculated for the population characteristics by FA dosage. Curve fitting by nonlinear regression was used to assess the adequate folic acid dose: mean relative changes in plasma tHcy concentration were plotted by dose of folic acid, and nonlinear regression was used to find the best-fit curve through the relative decreases in tHcy concentrations. In the exploratory analyses, a possible interaction with the *MTHFR* C677T genotype on the efficacy of folic acid and tHcy lowering was investigated. R software, version 2.15.1 (http://www.R-project.org/), was used for all statistical analyses.

## Results

### Baseline characteristics

**Fig. 1:**
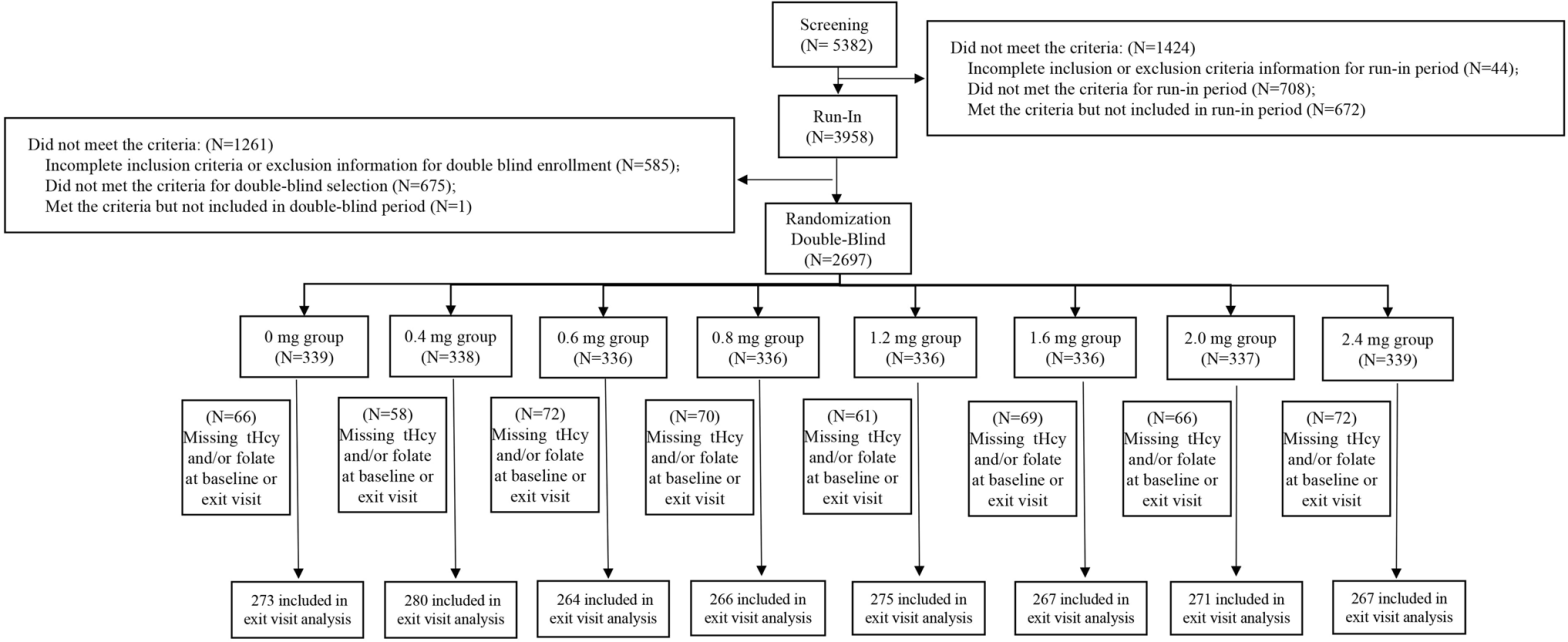
Design and flowchart of the study participants

A total of 2697 eligible participants were randomized to eight dosages of daily folate treatment groups. After excluding those with missing tHcy and/or folate data at either baseline or the exit visit (*n* = 534), 2163 eligible participants were included in the final analysis (Fig. 1). The baseline characteristics of the total population and by FA dosages are presented in Table 1, of which 45.4% were males (*n* = 981), mean age was 64.9 (SD 8.3) years, mean baseline tHcy level was 14.1 (11.9–17.5) µmol/L, and mean folate was 10.6 (7.2–15.3) ng/mL. These baseline characteristics indicate that a successful randomization within different treatment groups was carried out (Table 1).

**Table 1:** Baseline characteristics of participants in the Precision Folic Acid Trial to lower total homocysteine (tHcy)

Characteristics	Total	Folic acid treatment groups								*p* value
		0 mg	0.4 mg	0.6 mg	0.8 mg	1.2 mg	1.6 mg	2.0 mg	2.4 mg	
*N*	2163	273	280	264	266	275	267	271	267	
Males, no. (%)	981 (45.4%)	120 (44.0%)	128 (45.7%)	122 (46.2%)	118 (44.4%)	122 (44.4%)	118 (44.2%)	130 (48.0%)	123 (46.1%)	0.983
Age, year	64.9 ± 8.3	65.6 ± 8.2	64.2 ± 8.0	65.4 ± 8.4	65.1 ± 8.2	64.6 ± 8.3	64.7 ± 7.6	64.6 ± 8.6	64.9 ± 9.1	0.532
*BMI, kg/m*^*2*^	24.9 ± 5.0	25.0 ± 6.4	24.9 ± 3.5	25.3 ± 9.4	25.1 ± 3.7	24.6 ± 3.5	24.7 ± 3.5	24.7 ± 3.3	24.7 ± 3.4	0.714
SBP, mmHg	155.1 ± 17.8	156.2 ± 17.6	154.3 ± 17.0	155.1 ± 18.4	155.1 ± 16.5	154.0 ± 17.9	155.8 ± 17.1	155.3 ± 19.3	154.9 ± 18.4	0.885
DBP, mmHg	89.8 ± 10.9	89.7 ± 10.1	89.9 ± 11.1	89.9 ± 11.2	89.2 ± 9.7	88.9 ± 10.6	90.3 ± 11.0	90.2 ± 11.8	90.4 ± 11.2	0.682
Smoking status										0.864
Never	1352 (62.5%)	172 (63.0%)	179 (63.9%)	171 (64.8%)	164 (61.7%)	171 (62.2%)	170 (63.7%)	157 (57.9%)	168 (62.9%)	
Former	288 (13.3%)	36 (13.2%)	29 (10.4%)	38 (14.4%)	34 (12.8%)	36 (13.1%)	36 (13.5%)	46 (17.0%)	33 (12.4%)	
Current	523 (24.2%)	65 (23.8%)	72 (25.7%)	55 (20.8%)	68 (25.6%)	68 (24.7%)	61 (22.8%)	68 (25.1%)	66 (24.7%)	
Alcohol drinking status										0.955
Never	1101 (50.9%)	139 (50.9%)	135 (48.2%)	128 (48.5%)	136 (51.1%)	145 (52.7%)	141 (52.8%)	139 (51.3%)	138 (51.7%)	
Former	253 (11.7%)	39 (14.3%)	30 (10.7%)	32 (12.1%)	31 (11.7%)	28 (10.2%)	34 (12.7%)	30 (11.1%)	29 (10.9%)	
Current	809 (37.4%)	95 (34.8%)	115 (41.1%)	104 (39.4%)	99 (37.2%)	102 (37.1%)	92 (34.5%)	102 (37.6%)	100 (37.5%)	
MTHFR *genotypes, no. (%)*										0.997
CC	654 (30.2%)	84 (30.8%)	89 (31.8%)	79 (29.9%)	79 (29.7%)	86 (31.3%)	72 (27.0%)	78 (28.8%)	87 (32.6%)	
CT	991 (45.8%)	126 (46.2%)	128 (45.7%)	121 (45.8%)	125 (47.0%)	123 (44.7%)	125 (46.8%)	127 (46.9%)	116 (43.4%)	
TT	518 (23.9%)	63 (23.1%)	63 (22.5%)	64 (24.2%)	62 (23.3%)	66 (24.0%)	70 (26.2%)	66 (24.4%)	64 (24.0%)	
Center, no. (%)										0.999
Anqing	460 (21.3%)	59 (21.6%)	60 (21.4%)	57 (21.6%)	57 (21.4%)	58 (21.1%)	54 (20.2%)	55 (20.3%)	60 (22.5%)	
Lianyungang	870 (40.2%)	108 (39.6%)	107 (38.2%)	108 (40.9%)	109 (41.0%)	113 (41.1%)	109 (40.8%)	108 (39.9%)	108 (40.4%)	
Wuyuan	833 (38.5%)	106 (38.8%)	113 (40.4%)	99 (37.5%)	100 (37.6%)	104 (37.8%)	104 (39.0%)	108 (39.9%)	99 (37.1%)	
Laboratory results										
TC, mmol/L	5.4 ± 1.0	5.4 ± 1.1	5.3 ± 1.0	5.4 ± 1.0	5.3 ± 1.1	5.4 ± 1.0	5.4 ± 1.0	5.4 ± 1.1	5.4 ± 0.9	0.992
TG, mmol/L	1.4 (1.0–2.0)	1.3 (1.0–2.0)	1.4 (1.0–2.3)	1.4 (1.1–2.0)	1.4 (0.9–2.1)	1.4 (1.0–2.2)	1.4 (1.0–2.0)	1.4 (1.0–2.0)	1.4 (1.0–2.0)	0.817
HDL-C, mmol/L	1.7 ± 0.5	1.7 ± 0.4	1.7 ± 0.5	1.8 ± 0.5	1.7 ± 0.5	1.7 ± 0.5	1.7 ± 0.5	1.7 ± 0.4	1.7 ± 0.4	0.825
LDL-C, mmol/L	3.2 ± 0.8	3.2 ± 0.9	3.1 ± 0.8	3.1 ± 0.8	3.1 ± 0.8	3.2 ± 0.7	3.2 ± 0.8	3.2 ± 0.8	3.2 ± 0.7	0.893
FBG, mmol/L	6.0 ± 1.2	6.2 ± 1.4	5.9 ± 1.1	6.0 ± 1.1	6.0 ± 1.0	6.0 ± 1.1	6.0 ± 1.1	6.2 ± 1.3	5.9 ± 1.1	0.075
*eGFR, mL/min/1.73 m*^*2*^	88.5 ± 24.9	86.5 ± 25.5	89.3 ± 26.5	88.1 ± 21.1	89.4 ± 27.8	88.3 ± 23.3	89.1 ± 19.5	89.3 ± 29.2	88.0 ± 25.2	0.882
tHcy, μmol/L	14.1 (11.8–17.5)	14.4 (11.8–17.7)	14.1 (12.0–17.1)	14.7 (11.9–18.0)	13.8 (12.0–17.7)	13.3 (11.3–17.1)	14.3 (11.5–17.6)	14.2 (11.8–17.4)	14.2 (12.1–17.2)	0.280
Folate, ng/mL	10.6 (7.2–15.3)	11.5 (7.2–16.1)	10.3 (7.1–16.5)	10.3 (7.4–14.1)	10.1 (7.1–14.2)	10.7 (7.2–15.0)	10.8 (7.5–15.8)	10.6 (7.4–15.7)	10.6 (6.9–15.3)	0.564

Data are expressed as mean ± SD or median (interquartile range) and numbers (percentage) as appropriate

*BMI* body mass index, *SBP* systolic blood pressure, *DBP* diastolic blood pressure, *MTHFR* methylenetetrahydrofolate reductase, *TC* total cholesterol, *TG* triglycerides, *HDL-C* high-density lipoprotein cholesterol, *LDL-C* low-density lipoprotein cholesterol, *FBG* fasting blood glucose, *eGFR* estimated glomerular filtration rate, *tHcy* total homocysteine

### Percentage and absolute change of tHcy level reduction

Table 2 presents the absolute change value and percentage change of tHcy levels from baseline to the exit visit in the total population and in each FA treatment group stratified by *MTHFR C677T* genotypes. In the total population, with increasing doses of folate supplementation ranging from 0 to 2.4 mg, tHcy levels presented an overall decreasing association from 1.8 µmol/L in absolute change in tHcy levels for the 0.4 mg FA treatment group to 3.0 µmol/L in absolute change for the 2.4 FA treatment group. However, the greatest percentage of tHcy lowering was observed between the FA treatment groups 0.4–0.8 mg (change rate in tHcy: 7.4–9.9%) and in the 2.4 mg FA treatment group (change rate in tHcy: 11.2%).

**Table 2:** Changes in total homocysteine (tHcy) levels from baseline to exit visit by treatment groups in the total sample and in subgroups stratified by the MTHFR C677T genotypes

tHcy	Folic acid treatment groups							
	0 mg	0.4 mg	0.6 mg	0.8 mg	1.2 mg	1.6 mg	2.0 mg	2.4 mg
All participants								
At baseline [median (IQR), μmol/L]	14.4 (11.8–17.7)	14.1 (12.0–17.1)	14.7 (11.9–18.0)	13.8 (12.0–17.7)	13.3 (11.3–17.1)	14.3 (11.5–17.6)	14.2 (11.8–17.4)	14.2 (12.1–17.2)
At exit visit [median (IQR), μmol/L]	14.4 (11.7–18.8)	13.0 (10.8–16.2)	12.5 (10.4–15.6)	12.3 (10.4–15.2)	12.2 (10.1–14.8)	12.5 (10.4–15.9)	12.9 (10.2–15.9)	12.5 (10.6–15.7)
Absolute change [mean (95% CI), μmol/L]	− 0.6 (− 1.3, 0.2)	1.8 (1.1, 2.5)	2.1 (1.3, 2.8)	2.2 (1.4, 2.9)	1.9 (1.2, 2.6)	2.3 (1.5, 3.0)	2.0 (1.2, 2.7)	3.0 (2.3, 3.8)
Change rate [mean (95% CI), %]	− 4.6 (− 7.6, − 1.7)	7.4 (4.5, 10.3)	9.3 (6.3, 12.3)	9.9 (7.0, 12.9)	9.2 (6.3, 12.1)	8.7 (5.7, 11.6)	8.9 (6.0, 11.8)	11.2 (8.3, 14.2)
MTHFR C677T genotype								
CC								
At baseline [median (IQR), μmol/L]	13.8 (11.5–16.6)	13.7 (11.7–16.8)	14.0 (11.4–16.1)	13.6 (11.9–16.3)	12.6 (11.0–14.8)	13.6 (11.0–16.6)	13.5 (11.3–17.1)	13.5 (11.5–15.4)
At exit visit [median (IQR), μmol/L]	14.1 (11.7–19.0)	12.8 (10.9–15.4)	12.8 (10.4–15.3)	12.2 (10.4–14.6)	12.3 (9.9–14.2)	12.3 (9.7–15.6)	12.1 (10.2–15.7)	12.0 (10.7–14.8)
Absolute change [mean (95% CI), μmol/L]	− 1.2 (− 2.0, − 0.5)	0.7 (− 0.1, 1.5)	0.8 (− 0.0, 1.6)	1.6 (0.8, 2.5)	0.7 (− 0.1, 1.4)	1.3 (0.4, 2.1)	2.0 (1.2, 2.8)	1.4 (0.6, 2.2)
Change rate [mean (95% CI), %]	− 8.9 (− 13.7, − 4.1)	3.5 (− 1.2, 8.2)	4.8 (− 0.2, 9.7)	10.1 (5.1, 15.0)	3.9 (− 0.9, 8.7)	6.5 (1.3, 11.7)	9.0 (4.0, 14.0)	7.3 (2.6, 12.0)
CT								
At baseline [median (IQR), μmol/L]	14.0 (11.8–17.7)	14.1 (12.1–16.4)	14.6 (11.9–17.3)	13.4 (12.0–16.4)	13.9 (11.2–16.9)	13.9 (11.5–16.3)	13.7 (11.4–16.1)	13.8 (11.9–16.7)
At exit visit [median (IQR), μmol/L]	14.3 (11.2–17.5)	12.5 (10.5–15.6)	12.1 (10.2–15.5)	12.2 (10.8–15.2)	11.6 (10.0–13.9)	12.3 (10.4–14.7)	12.5 (10.1–15.3)	12.3 (10.2–15.6)
Absolute change [mean (95% CI), μmol/L]	− 0.1 (− 0.7, 0.6)	1.3 (0.7, 2.0)	1.2 (0.5, 1.9)	1.4 (0.7, 2.1)	2.0 (1.3, 2.7)	1.4 (0.7, 2.1)	1.4 (0.8, 2.1)	1.9 (1.2, 2.7)
Change rate [mean (95% CI), %]	− 0.9 (− 4.6, 2.8)	7.7 (4.0, 11.3)	8.4 (4.7, 12.2)	7.4 (3.8, 11.1)	11.8 (8.1, 15.5)	7.4 (3.7, 11.1)	8.6 (4.9, 12.2)	9.2 (5.4, 13.0)
TT								
At baseline [median (IQR), μmol/L]	15.0 (13.2–19.3)	15.3 (12.9–20.5)	16.9 (13.2–21.1)	15.5 (12.2–19.8)	14.4 (11.7–21.7)	17.0 (13.2–20.9)	16.1 (13.3–20.8)	16.8 (14.4–23.4)
At exit visit [median (IQR), μmol/L]	15.5 (12.2–20.4)	14.1 (11.5–18.6)	13.2 (11.1–17.9)	12.4 (10.2–17.1)	14.1 (10.8–17.6)	13.2 (11.1–17.4)	13.8 (10.9–17.1)	14.1 (11.7–16.7)
Absolute change [mean (95% CI), μmol/L]	− 0.7 (− 3.3, 1.8)	4.2 (1.6, 6.8)	5.2 (2.6, 7.8)	4.4 (1.8, 7.0)	3.4 (0.9, 5.9)	4.8 (2.4, 7.3)	3.0 (0.5, 5.5)	7.3 (4.7, 9.9)
Change rate [mean (95% CI), %]	− 6.5 (− 14.3, 1.3)	12.5 (4.6, 20.3)	16.5 (8.7, 24.2)	14.8 (6.9, 22.7)	11.4 (3.7, 19.0)	13.1 (5.7, 20.6)	9.4 (1.7, 17.0)	20.2 (12.4, 27.9)

When stratified by the *MTHFR* C677T genotypes CC, CT, and TT, an essentially higher tHcy-lowering effect was found in participants with the TT genotype and baseline tHcy > 15 μmol/L. With the increasing doses of folate supplementation from 0 to 2.4 mg, tHcy levels presented an overall decreasing association in all three genotype groups. The CC group showed the greatest fluctuations in the tHcy levels between the FA treatment doses of 1.2–1.6 mg, in the CT group between the FA doses 1.6–2.0 mg, and in the TT group between the FA doses 1.2–2.0 mg. The greatest tHcy-lowering rate was observed in the CC group at FA treatment 0.8 mg/day (tHcy lowering: 10.1%), in the CT group at 1.2 mg/day (tHcy lowering: 11.8%), and in the TT group at 2.4 mg/day (tHcy lowering: 20.2%).

**Fig. 2:**
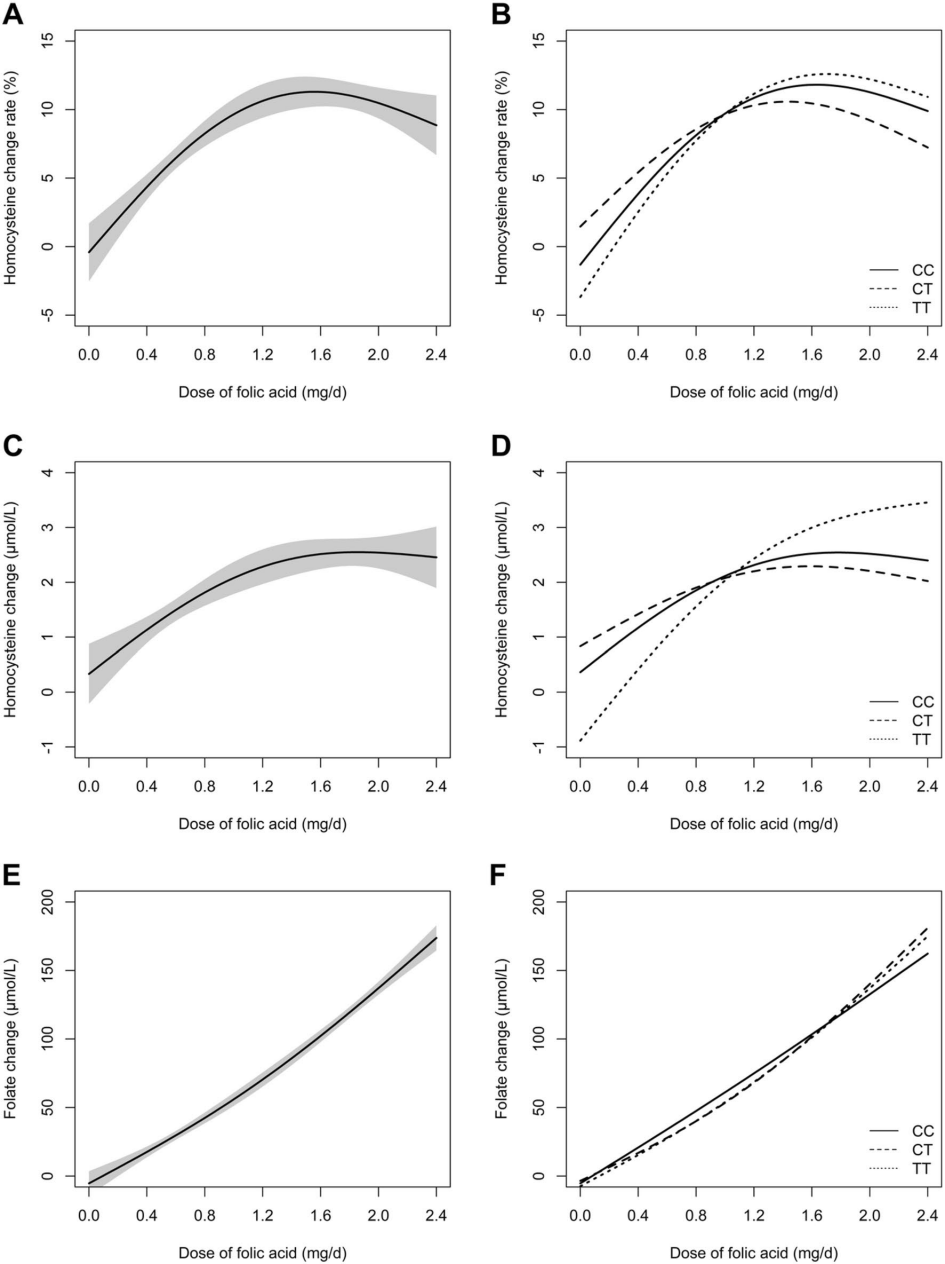
Adjusted fitted smoothing curves of serum total homocysteine (**A**–**D**) and folate (**E**, **F**) change and change rate for different doses of folic acid intervention at 8 weeks. **A** and **B** groups represent the change rate of total homocysteine in the overall population and subgroups stratified by MTHFR genotypes, respectively, across different doses of folic acid intervention at 8 weeks. **C** and **D** groups represent the absolute change of total homocysteine in the overall population and subgroups stratified by MTHFR genotypes, respectively. **E** and **F** groups represent the change of folate levels in the overall population and subgroups stratified by MTHFR genotypes, respectively, with varying folic acid dosage. Adjusted for age, sex, body mass index, systolic blood pressure, smoking status, center, high-density lipoprotein cholesterol, fasting blood glucose, *MTHFR* C677T, estimated glomerular filtration rate, and baseline folate level. Homocysteine and folate changes were defined as baseline − exit. Change rate were defined as (baseline − exit)/baseline)

Figure 2 illustrates the fitted smoothing curves of tHcy change rate/absolute value with doses of folate therapy in the total population (A and C) and stratified by the MTHFR genotypes (B and D) after 8 weeks of intervention after adjusting for sex, age, BMI, smoking status, systolic blood pressure (SBP), center, fasting blood glucose (FBG), high-density lipoprotein cholesterol (HDL-C), estimated glomerular filtration rate (eGFR), *MTHFR*, and folate levels. Changes/plateaus can be roughly observed between folic acid doses 1.2–1.6 mg/day in the total population.

Supplemental Tables 1 and 2 present the effects of different doses of folic acid treatment on tHcy change rates (Supplemental Table 1) and change values (Supplemental Table 2) in the total population and stratified by the *MTHFR* C677T genotype. In both tables, linear and quadratic associations between different doses of folic acid treatment with tHcy change rate and change in the total population were found to be significant after adjusting for covariates. The quadratic association presented a *β* value of − 4.48 (0.93)/− 0.62(0.24) for change rate and change, respectively, in the total population. The *P* value for joint effect (*df* = 2) was found to be significant (< 0.001). Similar results were identified in the *MTHFR* C677T stratified groups, with significant and negative *β* values in both the linear and quadratic associations between different doses of folic acid treatment with change rate/change value in tHcy levels.

### Changes in serum folate levels

Table 3 presents the change in serum folate levels from baseline to the exit visit for each treatment group in the total population and stratified by the *MTHFR* C677T genotypes. Both the total population and the genotype subgroups showed a dose-dependent increase in folic acid levels. Figure 2 shows the fitted smoothing curves of folate change with dose of folic acid in the total population (E) and stratified by the *MTHFR* C677T genotypes (F). These results are in accordance with the results in Table 3, showing that increases in folate change value is associated with a higher dose of folic acid treatment.

**Table 3:** Changes in serum folate from baseline to exit visit by treatment groups in the total sample and in subgroups stratified by the MTHFR C677T genotypes

Folate	Folic acid treatment groups							
	0 mg	0.4 mg	0.6 mg	0.8 mg	1.2 mg	1.6 mg	2.0 mg	2.4 mg
All participants								
At baseline [median (IQR), ng/mL]	11.5 (7.2–16.1)	10.3 (7.1–16.5)	10.3 (7.4–14.1)	10.1 (7.1–14.2)	10.7 (7.2–15.0)	10.8 (7.5–15.8)	10.6 (7.4–15.7)	10.6 (6.9–15.3)
At exit visit [median (IQR), ng/mL]	10.0 (7.0–14.8)	25.6 (16.1–39.2)	34.1 (17.9–52.9)	41.0 (19.2–72.3)	61.4 (21.7–137.8)	57.1 (19.6–176.8)	114.8 (26.6–275.8)	125.4 (27.2–300.1)
Change [mean (95% CI), ng/mL]	− 2.3 (− 13.9, 9.4)	16.3 (4.8, 27.7)	27.7 (15.9, 39.5)	39.4 (27.6, 51.1)	72.5 (61.0, 84.1)	97.7 (86.0, 109.5)	150.7 (139.1, 162.4)	165.8 (154.1, 177.6)
MTHFR C677T genotype								
CC								
At baseline [median (IQR), ng/mL]	13.0 (9.7–17.9)	12.1 (8.0–18.1)	11.5 (7.9–15.5)	11.4 (7.6–14.6)	11.7 (7.9–17.6)	12.1 (8.6–16.1)	11.2 (7.9–15.7)	12.2 (7.5–16.7)
At exit visit [median (IQR), ng/mL]	11.8 (7.8–16.3)	28.3 (18.4–39.8)	38.5 (20.5–52.2)	41.3 (19.7–77.0)	78.0 (29.1–131.2)	63.4 (21.7–170.8)	137.0 (29.2–266.8)	125.4 (31.7–286.9)
Change [mean (95% CI), ng/mL]	− 3.5 (− 22.7, 15.8)	13.0 (− 5.7, 31.7)	27.5 (7.6, 47.3)	43.3 (23.5, 63.1)	73.0 (54.0, 92.0)	94.3 (73.5, 115.0)	143.6 (123.6, 163.6)	151.3 (132.4, 170.3)
CT								
At baseline [median (IQR), ng/mL]	11.5 (7.4–16.5)	10.6 (7.3–16.6)	11.2 (8.1–16.0)	11.1 (7.2–15.2)	11.1 (7.1–15.2)	11.8 (8.5–16.9)	10.8 (7.5–16.5)	10.6 (7.2–16.4)
At exit visit [median (IQR), ng/mL]	10.0 (7.2–14.8)	25.2 (16.0–37.5)	39.5 (18.5–56.9)	44.4 (19.2–77.8)	54.0 (20.1–137.9)	55.4 (18.7–174.4)	131.5 (27.6–311.9)	99.0 (24.0–301.9)
Change [mean (95% CI), ng/mL]	− 1.3 (− 18.9, 16.4)	16.6 (− 0.9, 34.1)	31.6 (13.6, 49.6)	39.7 (22.0, 57.4)	67.3 (49.4, 85.2)	95.3 (77.6, 113.0)	165.5 (147.9, 183.0)	167.3 (148.9, 185.7)
TT								
At baseline [median (IQR), ng/mL]	8.7 (6.3–12.6)	7.5 (5.5–10.9)	8.0 (5.8–10.3)	7.7 (5.0–10.0)	9.1 (6.0–12.3)	8.3 (5.5–13.4)	8.9 (6.1–12.5)	8.0 (5.5–11.1)
At exit visit [median (IQR), ng/mL]	8.5 (6.2–11.1)	19.2 (13.4–38.4)	21.8 (14.4–39.4)	39.1 (17.8–64.2)	59.0 (20.5–147.4)	52.7 (20.2–194.2)	49.0 (20.1–258.7)	137.4 (41.0–317.1)
Change [mean (95% CI), ng/mL]	− 2.6 (− 27.7, 22.5)	20.3 (− 4.8, 45.4)	20.7 (− 4.2, 45.6)	33.6 (8.3, 58.9)	81.8 (57.3, 106.3)	105.6 (81.8, 129.4)	130.7 (106.2, 155.2)	182.9 (158.1, 207.8)

### Adverse events

The frequencies of reported adverse events, according to the Medical Dictionary for Regulatory Activities Primary System Organ Classification (SOC), and drug-related adverse events were not significantly different among the total sample (Table 4) or the subgroups stratified by the MTHFR C677T genotypes (Supplemental Tables 3–5). Furthermore, no statistical differences were found between the treatment groups with regard to other safety outcomes, such as adverse events leading to drug withdrawal, abnormal laboratory test results with clinical significance, and any serious adverse events.

**Table 4:** Safety profile by treatment groups in the total sample

All	Total		Folic acid treatment groups																*p* value
	(*N* = 2697)		0 mg (*N* = 339)		0.4 mg (*N* = 338)		0.6 mg (*N* = 336)		0.8 mg (*N* = 336)		1.2 mg (N = 336)		1.6 mg (*N* = 336)		2.0 mg (*N* = 337)		2.4 mg (*N* = 339)		
	Frequency	Participant, *N* (%)	Frequency	Participant, *N* (%)	Frequency	Participant, *N* (%)	Frequency	Participant, *N* (%)	Frequency	Participant, *N* (%)	Frequency	Participant, *N* (%)	Frequency	Participant, *N* (%)	Frequency	Participant, *N* (%)	Frequency	Participant, *N* (%)	
Respiratory, thoracic and mediastinal disorders	1355	894 (33.1)	184	115 (33.9)	174	119 (35.2)	150	100 (29.8)	169	118 (35.1)	170	108 (32.1)	169	110 (32.7)	177	122 (36.2)	162	102 (30.1)	0.529
Nervous system disorders	317	270 (10.0)	43	37 (10.9)	24	23 (6.8)	40	31 (9.2)	46	37 (11.0)	48	42 (12.5)	29	27 (8.0)	40	35 (10.4)	47	38 (11.2)	0.244
Gastrointestinal disorders	119	106 (3.9)	14	14 (4.1)	16	15 (4.4)	15	13 (3.9)	12	11 (3.3)	22	18 (5.4)	9	8 (2.4)	16	14 (4.2)	15	13 (3.8)	0.702
Abnormal laboratory test	61	60 (2.2)	9	8 (2.4)	6	6 (1.8)	7	7 (2.1)	10	10 (3.0)	11	11 (3.3)	5	5 (1.5)	8	8 (2.4)	5	5 (1.5)	0.697
Cardiac disorders	56	44 (1.6)	6	5 (1.5)	7	5 (1.5)	4	4 (1.2)	8	6 (1.8)	8	5 (1.5)	4	4 (1.2)	11	8 (2.4)	8	7 (2.1)	0.922
Renal and urinary disorders	49	48 (1.8)	7	7 (2.1)	8	8 (2.4)	2	2 (0.6)	4	4 (1.2)	7	7 (2.1)	8	7 (2.1)	5	5 (1.5)	8	8 (2.4)	0.616
General disorders and administration site conditions	38	37 (1.4)	6	5 (1.5)	4	4 (1.2)	4	4 (1.2)	8	8 (2.4)	7	7 (2.1)	2	2 (0.6)	3	3 (0.9)	4	4 (1.2)	0.522
Skin and subcutaneous tissue disorders	30	24 (0.9)	8	5 (1.5)	2	2 (0.6)	9	8 (2.4)	1	1 (0.3)	4	3 (0.9)	4	4 (1.2)	0	0 (0.0)	2	1 (0.3)	0.023
Eye disorders	21	21 (0.8)	2	2 (0.6)	3	3 (0.9)	2	2 (0.6)	6	6 (1.8)	2	2 (0.6)	1	1 (0.3)	4	4 (1.2)	1	1 (0.3)	0.362
Metabolism and nutrition disorders	19	19 (0.7)	3	3 (0.9)	3	3 (0.9)	3	3 (0.9)	1	1 (0.3)	3	3 (0.9)	3	3 (0.9)	2	2 (0.6)	1	1 (0.3)	0.927
Vascular and lymphatic diseases	18	18 (0.7)	4	4 (1.2)	2	2 (0.6)	1	1 (0.3)	2	2 (0.6)	3	3 (0.9)	2	2 (0.6)	3	3 (0.9)	1	1 (0.3)	0.852
Oral disease	13	13 (0.5)	2	2 (0.6)	0	0 (0.0)	0	0 (0.0)	2	2 (0.6)	0	0 (0.0)	2	2 (0.6)	3	3 (0.9)	4	4 (1.2)	0.202
Injury, poisoning and procedural complications	12	11 (0.4)	3	2 (0.6)	2	2 (0.6)	1	1 (0.3)	2	2 (0.6)	0	0 (0.0)	1	1 (0.3)	1	1 (0.3)	2	2 (0.6)	0.902
Musculoskeletal and connective tissue disorders	12	12 (0.4)	2	2 (0.6)	0	0 (0.0)	0	0 (0.0)	2	2 (0.6)	0	0 (0.0)	3	3 (0.9)	3	3 (0.9)	2	2 (0.6)	0.329
Hepatobiliary disorders	8	7 (0.3)	0	0 (0.0)	3	2 (0.6)	0	0 (0.0)	0	0 (0.0)	1	1 (0.3)	0	0 (0.0)	2	2 (0.6)	2	2 (0.6)	0.346
Endocrine disorders	6	5 (0.2)	3	2 (0.6)	1	1 (0.3)	0	0 (0.0)	0	0 (0.0)	1	1 (0.3)	0	0 (0.0)	0	0 (0.0)	1	1 (0.3)	0.520
Endocrine disorders	5	5 (0.2)	0	0 (0.0)	0	0 (0.0)	0	0 (0.0)	2	2 (0.6)	2	2 (0.6)	0	0 (0.0)	1	1 (0.3)	0	0 (0.0)	0.221
Infections and infestations	4	4 (0.1)	1	1 (0.3)	1	1 (0.3)	1	1 (0.3)	0	0 (0.0)	0	0 (0.0)	1	1 (0.3)	0	0 (0.0)	0	0 (0.0)	0.779
Reproductive system and breast disorders	3	3 (0.1)	1	1 (0.3)	0	0 (0.0)	0	0 (0.0)	0	0 (0.0)	1	1 (0.3)	1	1 (0.3)	0	0 (0.0)	0	0 (0.0)	0.659

### Sensitivity analysis

Supplemental Fig. 1 illustrates the fitted smoothing curves of tHcy change rate/absolute value with dose of folate therapy stratified by males and females (A and B) after 8 weeks of intervention with adjustment for age, sex, BMI, SBP, smoking status, center, HDL-C, FBG, *MTHFR*, eGFR, and folate levels. Changes/plateaus were roughly observed between folic acid doses 1.2–1.6 mg/day regardless of sex.

Supplemental Fig. 2 presents the adjusted smoothing curves of tHcy change (C and D) and the tHcy change rate (A and B) with doses of folic acid treatment in the total population (A and C), and for the *MTHFR* genotype stratified groups (B and D) with data from patients who had > 80% compliance (*n* = 1752). The results appear similar to the aforementioned results in Fig. 2.

## Discussion

This study investigated the interactive effect of the *MTHFR* C677T gene variant and serum folate levels on the tHcy-lowering response to short-term, varying doses of FA supplementation, in a population without mandatory FA fortification. To the best of our knowledge, this study is the first randomized, double-blinded trial to test the feasibility and effectiveness of different doses of FA supplementation for lowering tHcy and metabolites in the FA–tHcy metabolic pathway and the genetic interaction of the *MTHFR* C677T genotype in Chinese patients. This study found that after 8 weeks of folic acid supplementation, tHcy decreased by 9.2% in general. Combined with the results of CSPPT, which contains a similar population and found that 0.8 mg daily FA treatment increased folate levels and led to an average of tHcy levels reduction of 11%, these studies confirmed the stability of the current population.

This study explored a stable dose–effect relationship between 0 and 1.2 mg/day FA therapy on tHcy lowering. However, further increases in folate dose brought about a plateau. Wald et al. reported that serum tHcy levels decreased with increasing folic acid dosage and reached maximum efficacy at a dosage of 0.8 mg/day FA supplementation with a 23% reduction in tHcy among a population in Great Britain with ischemic heart disease [25]. A meta-analysis showed that a 1 mg/day folic acid dose generated the most tHcy-lowering efficacy, with no further reduction in tHcy levels with increasing dosages [21]. FA treatment ranging from 0.5 to 5.0 mg lowered tHcy levels by 25%. Studies that demonstrated promising results with reductions in tHcy levels have shown that the efficacy is more prominent among patients with high baseline tHcy levels or low folate levels before treatment [21]. A daily dose of approximately 400 μg is the minimum dose required for adequate tHcy reduction [26]. A 2010 study found that folic acid supplementation (0, 100, 400, 1000, or 2000 μg/day) had no dose–response relationship between FA and tHcy concentrations, but the data indicated that healthy, older adults (aged 60–90 years) can improve their folate status through supplementation [27].

This study found that the interaction between folic acid supplementation and tHcy exhibited distinct patterns for different *MTHFR* C677T genotypes among this population of Chinese hypertensive adults, along with different folic acid and tHcy levels at baseline across groups. In the TT group, lower doses of folic acid therapy (0.4–0.8 mg/day) were associated with maximum tHcy-lowering efficacy. The TT group had a steeper slope compared with the CC/CT group, and the fitted curve did not reach a plateau until 1.6 mg/day of folic acid supplement. The more effective tHcy-lowering capacity of folic acid therapy in the TT group can be explained by the fact that the highest baseline tHcy levels and the lowest folic acid levels were found in the TT group compared with the CC/CT group. The fitted curves for the correlation between folic acid treatment dose and changes in tHcy levels in different *MTHFR* C677T genotypes intersected at 1.0 mg/day of folic acid dose. In the CT group, the fitted curve reached a plateau at 1.0–1.2 mg/day of folic acid supplementation, and increased dose was found to be associated with a poorer tHcy reduction response. In the CC group, the fitted curve reached a plateau between 1.2 and 1.6 mg/day of folic acid supplement. The variability of tHcy reduction with folic acid treatment is in accordance with the results of the CSPPT, which determined an effect modification among the *MTHFR* genotypes in the efficacy of FA treatment. A post hoc analysis of the CSPPT found a more pronounced L-shaped curve between tHcy and serum folate levels in participants with the TT genotype compared with those with the CC and CT genotypes, requiring a higher folate level (at least 15 ng/mL) to eliminate genotypic tHcy differences. Our study results contribute to determining optimal folic acid intervention strategies in stroke risk prevention for hypertensive patients, especially in China where the effect of tHcy on first stroke is significantly modified by the methylenetetrahydrofolate reductase C677T genotype.

Our findings suggest a dose-dependent relationship between folate levels and folic acid supplementation. With increasing doses of folic acid supplementation, folate levels were elevated with no observed plateau, while the decreases in tHcy levels showed a significant plateau. The underlying mechanism can be explained by the differences in physical and chemical properties of synthetic folic acid. Folates are water-soluble vitamins that provide one-carbon units for the regulation of gene expression, nucleotide synthesis, and production of amino acids and neurotransmitters [28]. Available forms of synthetic folic acid include FA, folinic acid (formyl tetrahydrofolate), and 5-methyltetrahydrofolate (5-*MTHFR*). Folic acid from fortified foods and supplements differs from folate from natural sources, in that it requires dihydrofolate reductase (DHFR) for conversion to tetrahydrofolate to be active in one-carbon metabolism [29]. In areas that have FA fortification or synthetic FA recommendation [30, 31], circulating unmetabolized FA (UMFA) and 5-methyl-THF accounts for about 4% and 85% of total folate, respectively [32].

In a large national study with randomly selected US adults, both low and high serum total folates (total folate, UMFA, non-methyl folate, 5-mTHF, and MeFox [pyrazino-s-triazine derivative of 4ahydroxy-5-methyltetrahydrofolate]) were associated with a higher risk of all-cause, cardiovascular disease (CVD), and cause-specific mortality, including 5-mTHF insufficiency [33]. A study of major depressive disorder found that L-5-MeTHF improved symptoms in treatment-resistant major depressive disorder [34]. The MIREC (Maternal–Infant Research on Environmental Chemicals) study observed that the consumption of FA supplements by women resulted in a significantly increased total folate in breast milk. However, the increase in total milk folate was attributed to higher UMFA concentration, but not to reduced folates [35]. The higher proportion of UMFA in breast milk compared to 5-methylTHF in women consuming 400 μg FA daily suggests that higher doses exceed the physiological capacity to metabolize FA, resulting in preferential absorption of FA in breast milk. Our study population was characterized by low baseline levels of folate and with no FA fortification. The presence of a plateau in tHcy lowering within the range of 0.8–1.2 mg/day dose of folic acid therapy and the constant increase in folate levels for all doses indicate that caution should be taken for the use of higher folic acid doses for general population supplementation.

Folic acid therapy is frequently simultaneously considered with other B vitamins in metabolic cycles. The interpretation of clinical trial results for vitamin therapy to reduce tHcy levels is heavily reliant on folate levels, B12 status, and renal function [36, 37]. Most previous studies have adopted multiple vitamin B supplements with folate as the main component, for it has been shown that folate contributes the most to tHcy lowering, and additional supplements of B12 and B6 can lower tHcy levels by 7% [38].

No sex differences on the association between folic acid therapy and tHcy changes were observed in this study. A sensitivity analysis in the population with > 80% adherence showed the same patterns as the main study result.

Even in countries where fortified folic acid supplementation policies exist, most people still rely on food supplements, and a smaller proportion is aware of or adheres to the recommended daily intake levels [39]. Variation have been noted between countries in terms of the utilization, awareness, and beliefs toward FA supplement policies. Our study results highlighted the importance of evaluating and monitoring the utilization of supplements during antenatal care to facilitate appropriate usage. However, a systematic review demonstrated that in countries with mandatory food fortification policies, women who take FA supplements may surpass the upper tolerable limit of FA [40]. A recent study also found that excessive folic acid intake in parental mice increased DNA mutations and epigenetic changes in offspring embryos [41]. The problems of folic acid dosage, form, suitable population, and duration of supplementation have been plagued and are still ongoing.

Mammals, with the absence of folate biosynthesis, primarily meet their folate requirements through the diet. Historically, folate deficiency caused by poor nutrition has been one of the most widespread vitamin deficiencies and has persisted in countries that do not have mandatory FA fortification [42]. Future studies are critically needed to determine the optimal approaches in various pathological conditions. This study showed a precise dosage recommendation for a rural Chinese population with H-type hypertension, depending on the *MTHFR* C677T genotype.

This RCT has some limitations. The sample size was modest, although adequate power was expected based on the power estimation, for addressing the primary and secondary outcomes. This trial had only 8 weeks of treatment and follow-up. Although this duration is adequate for our primary and secondary outcomes, we were unable to evaluate long-term outcomes such as stroke incidence. The current analysis does not specifically confirm the proportion of people who might have actually met the target tHcy level window. Efforts are needed to try to increase the proportion of the whole population meeting the standards. To achieve a higher proportion of people with reduced levels, future analysis and research are required.

## Conclusion

In this population of rural Chinese adults with H-type hypertension, the optimal tHcy change rate corresponded to a folic acid dose range of 0–1.2 mg. A higher dose of FA had no effect on further lowering tHcy in the total population, with the exception of participants with the TT genotype. Further research is urgently needed to establish a safe and cost-effective FA regimen that is tailored to individual genetic profiles and folate nutritional status, to address stroke and CVD, which are major clinical and public health problems in China and many other developing countries.

### Supplementary Information

Below is the link to the electronic supplementary material.

Supplementary file1 (DOCX 2005 kb)

## Data Availability

The datasets used and/or analyzed in the current study are available from the corresponding author upon reasonable request.

## Abbreviations

FA: Folic acid
NTD: Neural-tube defects
tHcy: Total homocysteine
*MTHFR*: Methylenetetrahydrofolate reductase
5-MeTHF: 5-Methyltetrahydrofolate
BMI: Body mass index
SBP: Systolic blood pressure
HDL-C: High-density lipoprotein cholesterol
FBG: Fasting blood glucose
eGFR: Estimated glomerular filtration rate
UMFA: Unmetabolized folic acid
CVD: Cardiovascular disease
