# Supplementary material for: Optimal folic acid dosage in lowering homocysteine: Precision Folic Acid Trial to lower homocysteine (PFAT-Hcy)
Source: Eur J Nutr. 2024 Mar 13;63(5):1513–28. doi: 10.1007/s00394-024-03344-8 (PMC11329420; doi:10.1007/s00394-024-03344-8)
Supplement: Supplementary file 1 — Supplementary file1 (DOCX 2005 kb) [file 394_2024_3344_MOESM1_ESM.docx]

**Supplementary Materials**

**Optimal folic acid Dosage in lowering homocysteine: Precision Folic Acid Trial to lower homocysteine (PFAT-Hcy)**

Xiao Huang; Huihui Bao; Congcong Ding; Junpei Li; Tianyu Cao; Lishun Liu; Yaping Wei; Ziyi Zhou; Nan Zhang; Yun Song; Ping Chen; Chongfei Jiang; Liling Xie; Xianhui Qin; Yan Zhang; Jianping Li; Ningling Sun; Genfu Tang; Xiaobin Wang; Hong Wang; Yong Huo; Xiaoshu Cheng; for the Precision Folic Acid Trial to lower homocysteine (PFAT-Hcy) Trial Investigators

**Supplemental Table 1. Effects of different doses of folic acid on tHcy change rate in the total population and in subgroups stratified by the MTHFR C677T genotypes after 8 weeks of treatment.**

**Supplemental Table 2. Effects of different doses of folic acid on tHcy change in the total population and subgroups stratified by the MTHFR C677T genotypes after 8 weeks of treatment.**

**Supplemental Table 3. Safety profile by treatment groups in the CC group.**

**Supplemental Table 4. Safety profile by treatment groups in the CT group.**

**Supplemental Table 3. Safety profile by treatment groups in the TT group.**

**Supplemental Figure 1.** **Adjusted fitted smoothing curves of sex-stratified serum tHcy change (A) and change rate (B) for different doses of folic acid intervention at 8 weeks.** Adjusted for age, sex, body mass index, systolic blood pressure, smoking status, center, high-density lipoprotein cholesterol, fasting blood glucose, MTHFR C677T, estimated glomerular filtration rate, and baseline folate level.

**Supplemental Figure 2. Sensitivity analysis showing adjusted fitted smoothing curves of serum tHcy change (C and D) and change rate (A and B) for different doses of folic acid intervention at 8 weeks. Panels A, C for total sample and panels B, D for subgroups stratified by MTHFR genotypes. This analysis was limited to patients with compliance greater than 80%.** Adjusted for age, sex, body mass index, systolic blood pressure, smoking status, center, high-density lipoprotein cholesterol, fasting blood glucose, MTHFR C677T, estimated glomerular filtration rate, and baseline folate level. Homocysteine and folate change were defined as baseline – exit. Change rate were defined as (baseline-exit)/baseline).

**Supplemental Table 1. Effects of different doses of folic acid on tHcy change rate in the total population and in subgroups stratified by the MTHFR C677T genotypes after 8 weeks of treatment.**

|  | **N** | **Hcy change rate, Median (IQR)** | **Crude Model** | **Adjusted Model^a^** |
| --- | --- | --- | --- | --- |
|  |  |  | β (SE), P | β (SE), P |
| **All participants** | 2163 | 9.4 (-5.2-22.7) |  |  |
| **Linear** |  |  | 15.60 (2.54), <0.001 | 14.61 (2.35), <0.001 |
| **Quadratic** |  |  | -4.82 (1.01), <0.001 | -4.48 (0.93), <0.001 |
| P **for joint effect (df=2)** |  |  | <0.001 | <0.001 |
| **MTHFR C677T** |  |  |  |  |
| **CC** | 654 | 6.7 (-7.3-19.4) |  |  |
| **Linear** |  |  | 18.29 (4.22), <0.001 | 15.88 (3.91), <0.001 |
| **Quadratic** |  |  | -5.60 (1.66), <0.001 | -4.67 (1.54), 0.003 |
| P **for joint effect (df=2)** |  |  | <0.001 | <0.001 |
| **CT** | 991 | 9.3 (-4.8-21.5) |  |  |
| **Linear** |  |  | 12.14 (3.21), <0.001 | 12.33 (2.88), <0.001 |
| **Quadratic** |  |  | -3.96 (1.28), 0.002 | -4.14 (1.15), <0.001 |
| P **for joint effect (df=2)** |  |  | <0.001 | <0.001 |
| **TT** | 518 | 13.7 (-1.9-28.5) |  |  |
| **Linear** |  |  | 18.07 (6.77), 0.008 | 18.62 (6.39), 0.004 |
| **Quadratic** |  |  | -5.25 (2.68), 0.051 | -5.22 (2.53), 0.039 |
| P **for joint effect (df=2)** |  |  | 0.002 | <0.001 |
| P **for joint effect (CC vs TT)** |  |  | <0.001 | 0.015 |
| P **for joint effect (CT vs TT)** |  |  | 0.003 | 0.027 |

^a^Adjusted for age, sex, body mass index, systolic blood pressure, smoking status, center, high-density lipoprotein cholesterol, fasting blood glucose, MTHFR C677T, estimated glomerular filtration rate, and baseline folate level.

**Supplemental Table 2. Effects of different doses of folic acid on tHcy change in the total population and subgroups stratified by the MTHFR C677T genotypes after 8 weeks of treatment.**

|  | **N** | **Hcy change, Median (IQR)** | **Crude Model** | **Adjusted Model^a^** |
| --- | --- | --- | --- | --- |
|  |  |  | **β (SE), *P*** | **β (SE), *P*** |
| **All participants** | 2163 | 1.3 (-0.7-3.5) |  |  |
| **Linear** |  |  | 2.63 (0.65), <0.001 | 2.38 (0.61), <0.001 |
| **Quadratic** |  |  | -0.71 (0.26), 0.006 | -0.62 (0.24), 0.010 |
| ***P* for joint effect (df=2)** |  |  | <0.001 | <0.001 |
| **MTHFR C677T** |  |  |  |  |
| **CC** | 654 | 0.9 (-0.8-2.7) |  |  |
| **Linear** |  |  | 2.83 (0.69), <0.001 | 2.47 (0.65), <0.001 |
| **Quadratic** |  |  | -0.81 (0.27), 0.003 | -0.68 (0.26), 0.009 |
| ***P* for joint effect (df=2)** |  |  | <0.001 | <0.001 |
| **CT** | 991 | 1.2 (-0.7-3.2) |  |  |
| **Linear** |  |  | 1.76 (0.60), 0.003 | 1.77 (0.56), 0.002 |
| **Quadratic** |  |  | -0.51 (0.24), 0.033 | -0.53 (0.22), 0.017 |
| ***P* for joint effect (df=2)** |  |  | <0.001 | <0.001 |
| **TT** | 518 | 2.2 (-0.3-5.2) |  |  |
| **Linear** |  |  | 3.74 (2.22), 0.093 | 3.72 (2.08), 0.074 |
| **Quadratic** |  |  | -0.85 (0.88), 0.334 | -0.80 (0.82), 0.335 |
| ***P* for joint effect (df=2)** |  |  | 0.013 | 0.004 |
| ***P* for joint effect (CC vs TT)** |  |  | <0.001 | <0.001 |
| ***P* for joint effect (CT vs TT)** |  |  | <0.001 | <0.001 |

^a^Adjusted for age, sex, body mass index, systolic blood pressure, smoking status, center, high-density lipoprotein cholesterol, fasting blood glucose, MTHFR C677T, estimated glomerular filtration rate, and baseline folate level.

**Supplemental Table 3. Safety profile by treatment groups** **in the CC group.**

| **MTHFR C677T genotype = CC** | **Total** | | **Folic acid treatment groups** | | | | | | | | | | | | | | | | ***P*** *value* |
| --- | --- | --- | --- | --- | --- | --- | --- | --- | --- | --- | --- | --- | --- | --- | --- | --- | --- | --- | --- |
|  | **CC(N=846)** | | **0 mg(N=339)** | | **0.4 mg(N=338)** | | **0.6 mg(N=336)** | | **0.8 mg(N=336)** | | **1.2 mg(N=336)** | | **1.6 mg(N=336)** | | **2.0 mg(N=337)** | | **2.4 mg(N=339)** | |  |
|  | **Frequency** | **Participant，N(%)** | **Frequency** | **Participant，N(%)** | **Frequency** | **Participant，N(%)** | **Frequency** | **Participant，N(%)** | **Frequency** | **Participant，N(%)** | **Frequency** | **Participant，N(%)** | **Frequency** | **Participant，N(%)** | **Frequency** | **Participant，N(%)** | **Frequency** | **Participant，N(%)** |  |
| Respiratory, thoracic and mediastinal disorders | 382 | 266(31.4) | 52 | 33(30.6) | 60 | 42(39.6) | 30 | 23(22.1) | 43 | 31(29.2) | 48 | 30(28.6) | 55 | 37(35.2) | 53 | 43(40.6) | 41 | 27(25.5) | 0.04 |
| Nervous system disorders | 118 | 95(11.2) | 22 | 19(17.6) | 4 | 4(3.8) | 21 | 15(14.4) | 22 | 14(13.2) | 13 | 11(10.5) | 12 | 11(10.5) | 8 | 8(7.5) | 16 | 13(12.3) | 0.062 |
| Gastrointestinal disorders | 34 | 31(3.7) | 6 | 6(5.6) | 4 | 4(3.8) | 4 | 4(3.8) | 4 | 4(3.8) | 6 | 6(5.7) | 4 | 3(2.9) | 4 | 3(2.8) | 2 | 1(0.9) | 0.662 |
| Abnormal laboratory test | 11 | 11(1.3) | 1 | 1(0.9) | 1 | 1(0.9) | 1 | 1(1.0) | 1 | 1(0.9) | 5 | 5(4.8) | 0 | 0(0.0) | 1 | 1(0.9) | 1 | 1(0.9) | 0.107 |
| Cardiac disorders | 17 | 13(1.5) | 1 | 1(0.9) | 3 | 2(1.9) | 1 | 1(1.0) | 5 | 3(2.8) | 0 | 0(0.0) | 0 | 0(0.0) | 5 | 4(3.8) | 2 | 2(1.9) | 0.281 |
| Renal and urinary disorders | 16 | 15(1.8) | 3 | 3(2.8) | 3 | 3(2.8) | 1 | 1(1.0) | 1 | 1(0.9) | 4 | 4(3.8) | 2 | 1(1.0) | 0 | 0(0.0) | 2 | 2(1.9) | 0.435 |
| General disorders and administration site conditions | 15 | 15(1.8) | 0 | 0(0.0) | 3 | 3(2.8) | 2 | 2(1.9) | 3 | 3(2.8) | 4 | 4(3.8) | 0 | 0(0.0) | 0 | 0(0.0) | 3 | 3(2.8) | 0.172 |
| Skin and subcutaneous tissue disorders | 9 | 5(0.6) | 5 | 2(1.9) | 0 | 0(0.0) | 2 | 2(1.9) | 0 | 0(0.0) | 0 | 0(0.0) | 0 | 0(0.0) | 0 | 0(0.0) | 2 | 1(0.9) | 0.223 |
| Eye disorders | 12 | 12(1.4) | 1 | 1(0.9) | 1 | 1(0.9) | 1 | 1(1.0) | 5 | 5(4.7) | 0 | 0(0.0) | 1 | 1(1.0) | 2 | 2(1.9) | 1 | 1(0.9) | 0.149 |
| Metabolism and nutrition disorders | 7 | 7(0.8) | 1 | 1(0.9) | 1 | 1(0.9) | 2 | 2(1.9) | 0 | 0(0.0) | 1 | 1(1.0) | 1 | 1(1.0) | 1 | 1(0.9) | 0 | 0(0.0) | 0.848 |
| Vascular and lymphatic diseases | 7 | 7(0.8) | 1 | 1(0.9) | 1 | 1(0.9) | 0 | 0(0.0) | 0 | 0(0.0) | 2 | 2(1.9) | 1 | 1(1.0) | 1 | 1(0.9) | 1 | 1(0.9) | 0.854 |
| Oral disease | 4 | 4(0.5) | 0 | 0(0.0) | 0 | 0(0.0) | 0 | 0(0.0) | 0 | 0(0.0) | 0 | 0(0.0) | 1 | 1(1.0) | 2 | 2(1.9) | 1 | 1(0.9) | 0.33 |
| Injury, poisoning and procedural complications | 1 | 1(0.1) | 0 | 0(0.0) | 0 | 0(0.0) | 0 | 0(0.0) | 0 | 0(0.0) | 0 | 0(0.0) | 0 | 0(0.0) | 0 | 0(0.0) | 1 | 1(0.9) | 0.43 |
| Musculoskeletal and connective tissue disorders | 2 | 2(0.2) | 0 | 0(0.0) | 0 | 0(0.0) | 0 | 0(0.0) | 0 | 0(0.0) | 0 | 0(0.0) | 1 | 1(1.0) | 1 | 1(0.9) | 0 | 0(0.0) | 0.536 |
| Hepatobiliary disorders | 5 | 4(0.5) | 0 | 0(0.0) | 3 | 2(1.9) | 0 | 0(0.0) | 0 | 0(0.0) | 1 | 1(1.0) | 0 | 0(0.0) | 0 | 0(0.0) | 1 | 1(0.9) | 0.33 |
| Endocrine disorders | 3 | 2(0.2) | 2 | 1(0.9) | 0 | 0(0.0) | 0 | 0(0.0) | 0 | 0(0.0) | 0 | 0(0.0) | 0 | 0(0.0) | 0 | 0(0.0) | 1 | 1(0.9) | 0.549 |
| Endocrine disorders | 3 | 3(0.4) | 0 | 0(0.0) | 0 | 0(0.0) | 0 | 0(0.0) | 2 | 2(1.9) | 1 | 1(1.0) | 0 | 0(0.0) | 0 | 0(0.0) | 0 | 0(0.0) | 0.169 |
| Infections and infestations | 2 | 2(0.2) | 1 | 1(0.9) | 0 | 0(0.0) | 0 | 0(0.0) | 0 | 0(0.0) | 0 | 0(0.0) | 1 | 1(1.0) | 0 | 0(0.0) | 0 | 0(0.0) | 0.545 |

**Supplemental Table 4. Safety profile by treatment groups in the CT group.**

| **MTHFR C677T genotype = CT** | **Total** | | **Folic acid treatment groups** | | | | | | | | | | | | | | | | ***P value*** |
| --- | --- | --- | --- | --- | --- | --- | --- | --- | --- | --- | --- | --- | --- | --- | --- | --- | --- | --- | --- |
|  | **CT(N=1246)** | | **0 mg(N=339)** | | **0.4 mg(N=338)** | | **0.6 mg(N=336)** | | **0.8 mg(N=336)** | | **1.2 mg(N=336)** | | **1.6 mg(N=336)** | | **2.0 mg(N=337)** | | **2.4 mg(N=339)** | |  |
|  | **Frequency** | **Participant，N(%)** | **Frequency** | **Participant，N(%)** | **Frequency** | **Participant，N(%)** | **Frequency** | **Participant，N(%)** | **Frequency** | **Participant，N(%)** | **Frequency** | **Participant，N(%)** | **Frequency** | **Participant，N(%)** | **Frequency** | **Participant，N(%)** | **Frequency** | **Participant，N(%)** |  |
| Respiratory, thoracic and mediastinal disorders | 657 | 420(33.7) | 86 | 55(35.0) | 70 | 47(29.9) | 80 | 51(32.7) | 86 | 61(39.6) | 78 | 49(31.4) | 81 | 51(32.9) | 83 | 51(33.1) | 93 | 55(35.0) | 0.762 |
| Nervous system disorders | 126 | 113(9.1) | 14 | 13(8.3) | 11 | 10(6.4) | 9 | 7(4.5) | 16 | 15(9.7) | 21 | 18(11.5) | 12 | 11(7.1) | 26 | 23(14.9) | 17 | 16(10.2) | 0.049 |
| Gastrointestinal disorders | 58 | 50(4.0) | 5 | 5(3.2) | 6 | 5(3.2) | 7 | 5(3.2) | 7 | 6(3.9) | 15 | 11(7.1) | 3 | 3(1.9) | 7 | 7(4.5) | 8 | 8(5.1) | 0.44 |
| Abnormal laboratory test | 27 | 27(2.2) | 5 | 5(3.2) | 4 | 4(2.5) | 4 | 4(2.6) | 6 | 6(3.9) | 1 | 1(0.6) | 3 | 3(1.9) | 3 | 3(1.9) | 1 | 1(0.6) | 0.463 |
| Cardiac disorders | 25 | 20(1.6) | 3 | 2(1.3) | 2 | 2(1.3) | 3 | 3(1.9) | 2 | 2(1.3) | 4 | 2(1.3) | 2 | 2(1.3) | 4 | 3(1.9) | 5 | 4(2.5) | 0.978 |
| Renal and urinary disorders | 19 | 19(1.5) | 3 | 3(1.9) | 5 | 5(3.2) | 1 | 1(0.6) | 3 | 3(1.9) | 0 | 0(0.0) | 2 | 2(1.3) | 2 | 2(1.3) | 3 | 3(1.9) | 0.459 |
| General disorders and administration site conditions | 17 | 16(1.3) | 5 | 4(2.5) | 1 | 1(0.6) | 2 | 2(1.3) | 4 | 4(2.6) | 3 | 3(1.9) | 1 | 1(0.6) | 1 | 1(0.6) | 0 | 0(0.0) | 0.322 |
| Skin and subcutaneous tissue disorders | 17 | 15(1.2) | 2 | 2(1.3) | 2 | 2(1.3) | 6 | 5(3.2) | 1 | 1(0.6) | 3 | 2(1.3) | 3 | 3(1.9) | 0 | 0(0.0) | 0 | 0(0.0) | 0.18 |
| Eye disorders | 3 | 3(0.2) | 0 | 0(0.0) | 0 | 0(0.0) | 1 | 1(0.6) | 0 | 0(0.0) | 0 | 0(0.0) | 0 | 0(0.0) | 2 | 2(1.3) | 0 | 0(0.0) | 0.163 |
| Metabolism and nutrition disorders | 10 | 10(0.8) | 2 | 2(1.3) | 1 | 1(0.6) | 1 | 1(0.6) | 0 | 0(0.0) | 2 | 2(1.3) | 2 | 2(1.3) | 1 | 1(0.6) | 1 | 1(0.6) | 0.903 |
| Vascular and lymphatic diseases | 5 | 5(0.4) | 2 | 2(1.3) | 1 | 1(0.6) | 0 | 0(0.0) | 0 | 0(0.0) | 1 | 1(0.6) | 1 | 1(0.6) | 0 | 0(0.0) | 0 | 0(0.0) | 0.52 |
| Oral disease | 4 | 4(0.3) | 2 | 2(1.3) | 0 | 0(0.0) | 0 | 0(0.0) | 0 | 0(0.0) | 0 | 0(0.0) | 1 | 1(0.6) | 0 | 0(0.0) | 1 | 1(0.6) | 0.337 |
| Injury, poisoning and procedural complications | 7 | 6(0.5) | 2 | 1(0.6) | 1 | 1(0.6) | 0 | 0(0.0) | 2 | 2(1.3) | 0 | 0(0.0) | 1 | 1(0.6) | 1 | 1(0.6) | 0 | 0(0.0) | 0.69 |
| Musculoskeletal and connective tissue disorders | 8 | 8(0.6) | 1 | 1(0.6) | 0 | 0(0.0) | 0 | 0(0.0) | 1 | 1(0.6) | 0 | 0(0.0) | 2 | 2(1.3) | 2 | 2(1.3) | 2 | 2(1.3) | 0.531 |
| Hepatobiliary disorders | 1 | 1(0.1) | 0 | 0(0.0) | 0 | 0(0.0) | 0 | 0(0.0) | 0 | 0(0.0) | 0 | 0(0.0) | 0 | 0(0.0) | 1 | 1(0.6) | 0 | 0(0.0) | 0.419 |
| Endocrine disorders | 1 | 1(0.1) | 0 | 0(0.0) | 0 | 0(0.0) | 0 | 0(0.0) | 0 | 0(0.0) | 1 | 1(0.6) | 0 | 0(0.0) | 0 | 0(0.0) | 0 | 0(0.0) | 0.43 |
| Endocrine disorders | 1 | 1(0.1) | 0 | 0(0.0) | 0 | 0(0.0) | 0 | 0(0.0) | 0 | 0(0.0) | 1 | 1(0.6) | 0 | 0(0.0) | 0 | 0(0.0) | 0 | 0(0.0) | 0.43 |
| Infections and infestations | 1 | 1(0.1) | 0 | 0(0.0) | 1 | 1(0.6) | 0 | 0(0.0) | 0 | 0(0.0) | 0 | 0(0.0) | 0 | 0(0.0) | 0 | 0(0.0) | 0 | 0(0.0) | 0.435 |

**Supplemental Table 5. Safety profile by treatment groups in the TT group.**

| **MTHFR C677T genotype = TT** | **Total** | | **Folic acid treatment groups** | | | | | | | | | | | | | | | | ***P value*** |
| --- | --- | --- | --- | --- | --- | --- | --- | --- | --- | --- | --- | --- | --- | --- | --- | --- | --- | --- | --- |
|  | **TT(N=605)** | | **0 mg(N=339)** | | **0.4 mg(N=338)** | | **0.6 mg(N=336)** | | **0.8 mg(N=336)** | | **1.2 mg(N=336)** | | **1.6 mg(N=336)** | | **2.0 mg(N=337)** | | **2.4 mg(N=339)** | |  |
|  | **Frequency** | **Participant，N(%)** | **Frequency** | **Participant，N(%)** | **Frequency** | **Participant，N(%)** | **Frequency** | **Participant，N(%)** | **Frequency** | **Participant，N(%)** | **Frequency** | **Participant，N(%)** | **Frequency** | **Participant，N(%)** | **Frequency** | **Participant，N(%)** | **Frequency** | **Participant，N(%)** |  |
| Respiratory, thoracic and mediastinal disorders | 316 | 208(34.4) | 46 | 27(36.5) | 44 | 30(40.0) | 40 | 26(34.2) | 40 | 26(34.2) | 44 | 29(38.7) | 33 | 22(28.9) | 41 | 28(36.4) | 28 | 20(26.3) | 0.644 |
| Nervous system disorders | 73 | 62(10.2) | 7 | 5(6.8) | 9 | 9(12.0) | 10 | 9(11.8) | 8 | 8(10.5) | 14 | 13(17.3) | 5 | 5(6.6) | 6 | 4(5.2) | 14 | 9(11.8) | 0.253 |
| Gastrointestinal disorders | 27 | 25(4.1) | 3 | 3(4.1) | 6 | 6(8.0) | 4 | 4(5.3) | 1 | 1(1.3) | 1 | 1(1.3) | 2 | 2(2.6) | 5 | 4(5.2) | 5 | 4(5.3) | 0.431 |
| Abnormal laboratory test | 23 | 22(3.6) | 3 | 2(2.7) | 1 | 1(1.3) | 2 | 2(2.6) | 3 | 3(3.9) | 5 | 5(6.7) | 2 | 2(2.6) | 4 | 4(5.2) | 3 | 3(3.9) | 0.745 |
| Cardiac disorders | 14 | 11(1.8) | 2 | 2(2.7) | 2 | 1(1.3) | 0 | 0(0.0) | 1 | 1(1.3) | 4 | 3(4.0) | 2 | 2(2.6) | 2 | 1(1.3) | 1 | 1(1.3) | 0.728 |
| Renal and urinary disorders | 14 | 14(2.3) | 1 | 1(1.4) | 0 | 0(0.0) | 0 | 0(0.0) | 0 | 0(0.0) | 3 | 3(4.0) | 4 | 4(5.3) | 3 | 3(3.9) | 3 | 3(3.9) | 0.126 |
| General disorders and administration site conditions | 6 | 6(1.0) | 1 | 1(1.4) | 0 | 0(0.0) | 0 | 0(0.0) | 1 | 1(1.3) | 0 | 0(0.0) | 1 | 1(1.3) | 2 | 2(2.6) | 1 | 1(1.3) | 0.705 |
| Skin and subcutaneous tissue disorders | 4 | 4(0.7) | 1 | 1(1.4) | 0 | 0(0.0) | 1 | 1(1.3) | 0 | 0(0.0) | 1 | 1(1.3) | 1 | 1(1.3) | 0 | 0(0.0) | 0 | 0(0.0) | 0.772 |
| Eye disorders | 6 | 6(1.0) | 1 | 1(1.4) | 2 | 2(2.7) | 0 | 0(0.0) | 1 | 1(1.3) | 2 | 2(2.7) | 0 | 0(0.0) | 0 | 0(0.0) | 0 | 0(0.0) | 0.377 |
| Metabolism and nutrition disorders | 2 | 2(0.3) | 0 | 0(0.0) | 1 | 1(1.3) | 0 | 0(0.0) | 1 | 1(1.3) | 0 | 0(0.0) | 0 | 0(0.0) | 0 | 0(0.0) | 0 | 0(0.0) | 0.536 |
| Vascular and lymphatic diseases | 6 | 6(1.0) | 1 | 1(1.4) | 0 | 0(0.0) | 1 | 1(1.3) | 2 | 2(2.6) | 0 | 0(0.0) | 0 | 0(0.0) | 2 | 2(2.6) | 0 | 0(0.0) | 0.398 |
| Oral disease | 5 | 5(0.8) | 0 | 0(0.0) | 0 | 0(0.0) | 0 | 0(0.0) | 2 | 2(2.6) | 0 | 0(0.0) | 0 | 0(0.0) | 1 | 1(1.3) | 2 | 2(2.6) | 0.226 |
| Injury, poisoning and procedural complications | 4 | 4(0.7) | 1 | 1(1.4) | 1 | 1(1.3) | 1 | 1(1.3) | 0 | 0(0.0) | 0 | 0(0.0) | 0 | 0(0.0) | 0 | 0(0.0) | 1 | 1(1.3) | 0.772 |
| Musculoskeletal and connective tissue disorders | 2 | 2(0.3) | 1 | 1(1.4) | 0 | 0(0.0) | 0 | 0(0.0) | 1 | 1(1.3) | 0 | 0(0.0) | 0 | 0(0.0) | 0 | 0(0.0) | 0 | 0(0.0) | 0.529 |
| Hepatobiliary disorders | 2 | 2(0.3) | 0 | 0(0.0) | 0 | 0(0.0) | 0 | 0(0.0) | 0 | 0(0.0) | 0 | 0(0.0) | 0 | 0(0.0) | 1 | 1(1.3) | 1 | 1(1.3) | 0.548 |
| Endocrine disorders | 2 | 2(0.3) | 1 | 1(1.4) | 1 | 1(1.3) | 0 | 0(0.0) | 0 | 0(0.0) | 0 | 0(0.0) | 0 | 0(0.0) | 0 | 0(0.0) | 0 | 0(0.0) | 0.523 |
| Endocrine disorders | 1 | 1(0.2) | 0 | 0(0.0) | 0 | 0(0.0) | 0 | 0(0.0) | 0 | 0(0.0) | 0 | 0(0.0) | 0 | 0(0.0) | 1 | 1(1.3) | 0 | 0(0.0) | 0.443 |
| Infections and infestations | 1 | 1(0.2) | 0 | 0(0.0) | 0 | 0(0.0) | 1 | 1(1.3) | 0 | 0(0.0) | 0 | 0(0.0) | 0 | 0(0.0) | 0 | 0(0.0) | 0 | 0(0.0) | 0.432 |

**
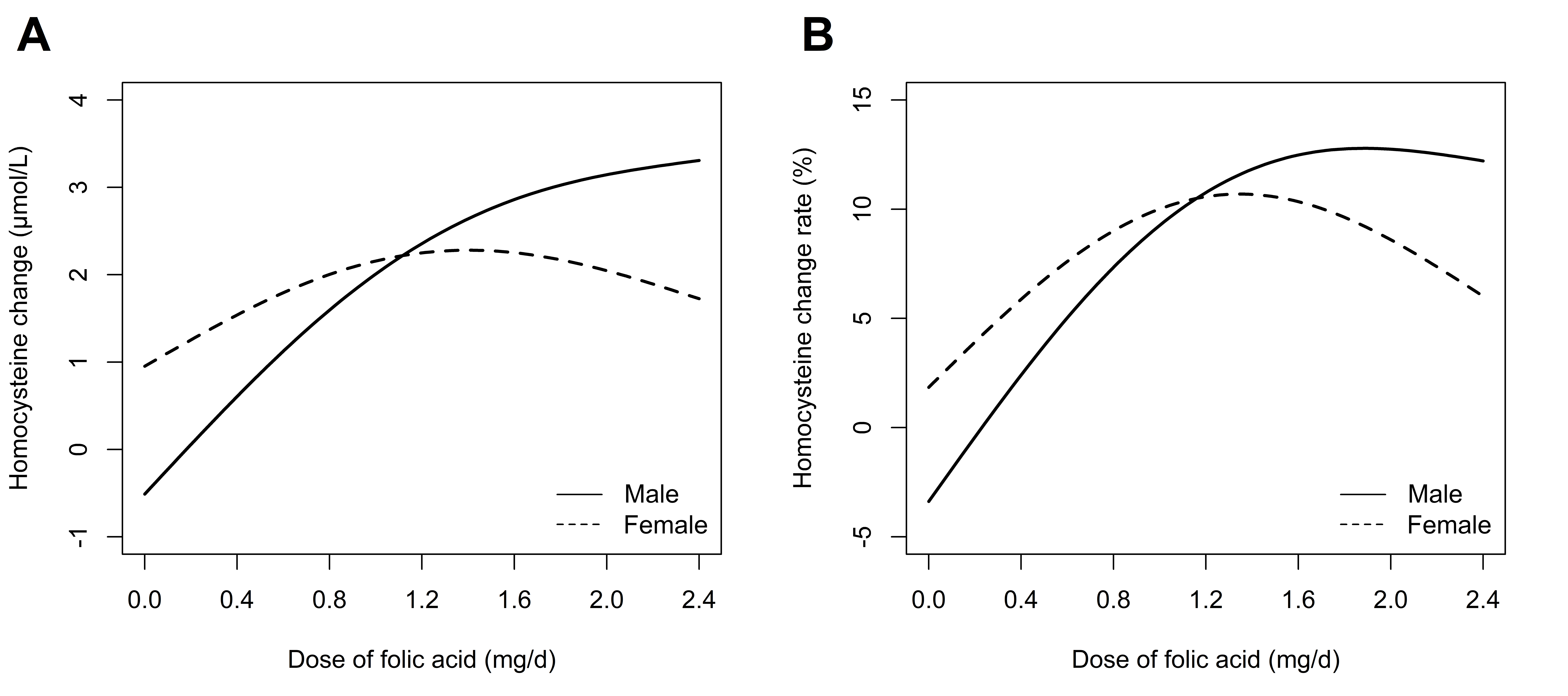
**

**Supplemental Figure 1.** **Adjusted fitted smoothing curves of sex-stratified serum tHcy change (A) and change rate (B) for different doses of folic acid intervention at 8 weeks.** Adjusted for age, sex, body mass index, systolic blood pressure, smoking status, center, high-density lipoprotein cholesterol, fasting blood glucose, MTHFR C677T, estimated glomerular filtration rate, and baseline folate level.





**Supplemental Figure 2. Sensitivity analysis showing** **adjusted fitted smoothing curves of serum tHcy change (C and D) and change rate (A and B) for different doses of folic acid intervention at 8 weeks. Panels A, C for total sample and panels B, D for subgroups stratified by MTHFR genotypes. This analysis was limited to patients with compliance greater than 80%.** Adjusted for age, sex, body mass index, systolic blood pressure, smoking status, center, high-density lipoprotein cholesterol, fasting blood glucose, MTHFR C677T, estimated glomerular filtration rate, and baseline folate level. Homocysteine and folate change were defined as baseline – exit. Change rate were defined as (baseline-exit)/baseline).
